# Open-access programs for injury categorization using ICD-9 or ICD-10

**DOI:** 10.1186/s40621-018-0149-8

**Published:** 2018-04-09

**Authors:** David E. Clark, Adam W. Black, David H. Skavdahl, Lee D. Hallagan

**Affiliations:** 1 Department of Surgery, Maine Medical Center, Portland, ME USA; 2 MMC Center for Outcomes Research and Evaluation, Maine Medical Center, 509 Forest Avenue, Portland, ME 04101 USA; 3 Tufts University School of Medicine, Boston, MA USA

**Keywords:** Injury severity score, Abbreviated injury score, International classification of diseases, ICDPIC

## Abstract

**Background:**

The article introduces Programs for Injury Categorization, using the International Classification of Diseases (ICD) and R statistical software (ICDPIC-R). Starting with ICD-8, methods have been described to map injury diagnosis codes to severity scores, especially the Abbreviated Injury Scale (AIS) and Injury Severity Score (ISS). ICDPIC was originally developed for this purpose using Stata, and ICDPIC-R is an open-access update that accepts both ICD-9 and ICD-10 codes.

**Methods:**

Data were obtained from the National Trauma Data Bank (NTDB), Admission Year 2015. ICDPIC-R derives CDC injury mechanism categories and an approximate ISS (“*RISS*”) from either ICD-9 or ICD-10 codes. For ICD-9-coded cases, *RISS* is derived similar to the Stata package (with some improvements reflecting user feedback). For ICD-10-coded cases, *RISS* may be calculated in several ways: The “GEM” methods convert ICD-10 to ICD-9 (using General Equivalence Mapping tables from CMS) and then calculate ISS with options similar to the Stata package; a “ROCmax” method calculates *RISS* directly from ICD-10 codes, based on diagnosis-specific mortality in the NTDB, maximizing the C-statistic for predicting NTDB mortality while attempting to minimize the difference between *RISS* and ISS submitted by NTDB registrars (*ISSAIS*). Findings were validated using data from the National Inpatient Survey (NIS, 2015).

**Results:**

NTDB contained 917,865 cases, of which 86,878 had valid ICD-10 injury codes. For a random 100,000 ICD-9-coded cases in NTDB, *RISS* using the GEM methods was nearly identical to ISS calculated by the Stata version, which has been previously validated. For ICD-10-coded cases in NTDB, categorized ISS using any version of *RISS* was similar to *ISSAIS;* for both NTDB and NIS cases, increasing ISS was associated with increasing mortality. Prediction of NTDB mortality was associated with C-statistics of 0.81 for *ISSAIS*, 0.75 for *RISS* using the GEM methods, and 0.85 for *RISS* using the ROCmax method; prediction of NIS mortality was associated with C-statistics of 0.75–0.76 for *RISS* using the GEM methods, and 0.78 for *RISS* using the ROCmax method. Instructions are provided for accessing ICDPIC-R at no cost.

**Conclusions:**

The ideal methods of injury categorization and injury severity scoring involve trained personnel with access to injured persons or their medical records. ICDPIC-R may be a useful substitute when this ideal cannot be obtained.

## Introduction

This article describes Programs for Injury Categorization, using the International Classification of Diseases (ICD) and R statistical software (ICDPIC-R). These programs are intended to provide inexpensive methods for translating ICD injury diagnosis codes (either the Ninth or Tenth Revision) into standard categories and/or scores. The popular open-source statistical program R (R Foundation for Statistical Computing, Vienna, Austria) is used as a data management and programming platform, and must be separately obtained (at no charge) in order to run the programs.

The programming code and associated tables for ICDPIC-R are completely open-source, are provided as a public service, and may be used without charge. The authors will appreciate having this article cited if the software is used for other studies. We hope ICDPIC-R will facilitate the use of ICD codes for epidemiologic or health services research about injured persons.

## Background

### Injury severity scoring from anatomic injury codes

Administrative databases and registries often record specific injuries using the ICD, which has gone from its Eighth Revision (ICD-8) through its Ninth (ICD-9) and Tenth (ICD-10) Revisions over the past few decades (with an Eleventh Revision already being planned). More detailed modifications of the ICD systems have been developed in different countries, and most administrative data in the United States are now coded using a “Clinical Modification” of the Tenth Revision (ICD-10-CM). There have been numerous prior efforts to convert ICD codes into measures of injury severity.

The Abbreviated Injury Scale (AIS) (Committee on Medical Aspects of Automotive Safety, AMA, 1971) was among the earliest attempts to categorize injuries by body region and severity, although it was not initially defined in terms of ICD diagnoses. The original version of the AIS, developed and published by the American Medical Association (AMA) in 1971 (Committee on Medical Aspects of Automotive Safety, AMA, 1971), is used as a basis for ICDPIC-R. Later versions of the AIS, with different body regions and revised definitions, have been produced and copyrighted by the Association for the Advancement of Automotive Medicine (AAAM, Chicago IL), and are available from that organization for a fee. ICDPIC-R does not use these newer versions of the AIS.

Soon after the original publication of the AIS, Baker and colleagues extended it to create an Injury Severity Score (ISS) (Baker et al., 1974), which has subsequently been the most common way to describe injury severity, and indeed theirs is one of the most frequently cited publications in the surgical literature (Paladugu et al., 2002). Under ICD-8, American hospital data were recorded using an “ICD, Adapted” (ICDA) classification, and the initial development of the ISS included a mapping by which “each ICDA code was translated into an AIS grade” (Baker et al., 1974).

Other methods based on the AIS have been described, including the Anatomic Profile (AP) (Copes et al., 1990), the New Injury Severity Score (NISS) (Osler et al., 1997), and the Trauma Mortality Prediction Model (TMPM) (Osler et al., 2008), but these have not replaced the ISS in popular use.

Some investigators have bypassed the AIS to derive severity scores directly from ICD codes and objective outcomes in a large database. One drawback of this approach is that the data to implement this approach are generally available only for hospital inpatients and therefore do not include the large proportion of injury deaths that occur without hospital admission. Levy and colleagues (Levy et al., 1978) tabulated the observed hospital survival for patients with each ICDA injury diagnosis code, and assigned an Estimated Survival Probability (ESP) equal to the product of the observed survival probabilities for each patient. Osler and colleagues later published a method similar to the ESP and named it the ICD-9-based Injury Severity Score (ICISS) (Osler et al., 1996). Barell and colleagues (Barell et al., 2002) described a matrix to classify ICD-9 injury diagnoses by type and anatomic region, and this matrix can be combined with objective data to produce a severity score (Clark & Ahmad, 2006). The TMPM can also be calculated directly from ICD-9-CM codes (Glance et al., 2009).

### Combining other data with anatomic severity scores

The Trauma and Injury Severity Score (TRISS) (Champion & Sacco, 1983; Boyd et al., 1987) derived survival predictions for blunt or penetrating trauma from a combination of ISS, age, and physiologic data. A Severity Characterization of Trauma (ASCOT) (Champion et al., 1990) similarly derived survival predictions for blunt or penetrating trauma from a combination of AP, age, and physiologic data. Neither TRISS nor ASCOT is used much today, but many authors have noted the increased accuracy when anatomic injury descriptions are combined with other predictive factors.

Beyond the distinction between blunt and penetrating injury, more specific classifications of injury mechanism can be incorporated into predictive models of outcome after injury. External Cause of Injury codes in ICD-9 (E-codes) refer to the mechanism of injury, such as vehicle crash or gunshot. ICD-10 also includes codes describing External Cause of Injury (codes starting with U, V, W, X, or Y). The CDC has published a recommended categorization of ICD-9 E-codes by mechanism (CDC, 1997), and has proposed a similar categorization of ICD-10 codes related to injury mechanism (Annest et al., 2014).

Beyond age, other measures of underlying health can also be incorporated into predictive models of outcome after injury. One common measure of comorbidity is the weighted score derived by Charlson et al. (Charlson et al., 1987), which has been modified for use with ICD-9-CM data using the methods of Romano et al. (Romano et al., 1993) or Deyo et al. (Deyo et al., 1994) Another comorbidity score based directly on ICD-9-CM codes was developed by Elixhauser et al. (Elixhauser et al., 1998) The Charlson-Deyo and Elixhauser comorbidity scores have been updated for ICD-10-CM by Quan et al. (Quan et al., 2005)

Physiologic data are not generally coded in ICD-9 or ICD-10. However, they are often recorded in trauma registries, certainly have prognostic value, and can be combined with anatomic, mechanism, comorbidity, and other data for outcome prediction or risk adjustment.

### Software mapping anatomic ICD codes to severity scores

A program to standardize the conversion of ICD-9-CM to AIS was developed with federal grant support at the Johns Hopkins School of Hygiene and Public Health (MacKenzie et al., 1989), and was subsequently marketed as ICDMAP-90 by the Tri-Analytics Corporation (Bel Air, Maryland). However, ICDMAP-90 required payment of a fee, used proprietary algorithms, and did not recognize some more recent ICD-9-CM codes (or ICD-10-CM codes).

Recognizing the limitations of ICDMAP-90, Clark and colleagues developed ICDPIC in 2008 using Stata (StataCorp, College Station TX) (Clark et al., 2010). ICDPIC (now in Version 3) is freely available and open-source, but requires that the user have a copy of Stata. ICDPIC reads standardized administrative data and will calculate AIS, ISS, AP, NISS, TRISS, ASCOT, ICISS, TMPM, Barell Matrix, CDC mechanism categories, and Charlson-Romano or Elixhauser comorbidity scores, but is limited to data coded using ICD-9-CM.

Despite the ability of ICDPIC to calculate numerous severity scores of historical interest, some of them arguably more accurate than ISS for hospitalized patients (Van Belleghem et al., 2016; Meredith et al., 2002), researchers have primarily used it to obtain an approximate AIS and/or ISS. The initial methodology for ICDPIC to perform this function was developed using ICD-9-CM codes and ISS data from the 2008 American College of Surgeons (ACS) National Trauma Data Bank (NTDB), and it has been validated by several independent researchers (Sears et al., 2014; Greene et al., 2015; Fleischman et al., 2017). Mapping from ICD codes will obviously not be as accurate as AIS or ISS scoring based on direct access to the actual person or primary medical record (Meredith et al., 2002; Di Bartolomeo et al., 2010), and might be more accurate if more recent versions of the AIS were applied, but it appears to be a reasonable substitute when other methods are impractical or unavailable.

The arrival of ICD-10 led to the development of subjective AIS mapping methods in Canada (Haas et al., 2012), Germany (Hartensuer et al., 2015), and probably elsewhere, but software to implement these methods has not been made public and may be specific to the versions of ICD-10 used in those countries. More recently, a panel of experts convened by the AAAM has developed a mapping of the U.S. version of ICD-10-CM to AIS (Loftis et al., 2016), and its website describes the licensing agreements and fees required for its use.

There has been an ongoing debate about whether injury severity should be predicted based on expert opinion or derived from a reference database (Van Belleghem et al., 2016; Committee on Trauma, ACS, 2017). The former approach has generally been used for AIS and ISS, but the development of ICDPIC-R provides an opportunity to combine the familiar ISS format with a data-driven approach. This may allow for improved prediction of mortality for patient populations similar to the reference database, although the subjective versus objective methodologic debate is likely to continue.

## Methods

ICDPIC-R is intended to be freely available for any user, and accordingly has been implemented using the open-source statistical software R (R Foundation for Statistical Computing, Vienna, Austria). For development and testing purposes, the National Trauma Data Bank (NTDB) research data set for admission year 2015 was obtained from the American College of Surgeons (ACS) (Hedegaard et al., 2016) in compliance with its standard Data Use Agreement. For purposes of validation, the National Inpatient Sample (NIS) data sets for the Fourth Quarter of 2015 were also obtained from the Agency for Healthcare Research and Quality (AHRQ) in compliance with its standard Data Use Agreement.

For each valid ICD-9-CM or ICD-10-CM injury diagnosis, ICDPIC-R is programmed to generate an approximate AIS and body region, using the original AIS anatomic classification (as modified by Baker and colleagues) into six body regions: Head and neck, face, chest, abdomen and pelvic contents, extremities and pelvic bones, and general (Committee on Medical Aspects of Automotive Safety, AMA, 1971; Baker et al., 1974). In addition, each code referring to a mechanism of injury is categorized as recommended or proposed by the CDC (CDC, 1997; Annest et al., 2014). For each injured person, ICDPIC-R determines the maximal AIS in each body region and overall, an Injury Severity Score (*RISS*), and a CDC mechanism category.

Mapping of ICD-9-CM codes to AIS severity and body region utilizes essentially the same table that was used for the Stata implementation of ICDPIC. After initial testing, we reclassified code 850.11 to AIS = 2 as recommended by Fleischman et al. (Fleischman et al., 2017), and codes 806.1 and 862.8 to AIS = 5 as recommended by DiBartolomeo et al. (Di Bartolomeo et al., 2010)

Mapping of ICD-9-CM E-codes to CDC mechanism categories simply involved translation of the programming code from Stata into R, using the same table. Mapping of ICD-10-CM codes to mechanism categories was based on a similar table published by the CDC (Annest et al., 2014).

The National Trauma Data Standard used by NTDB considers valid ICD-10-CM injury codes to be those in the ranges S00-S99, T07, T14, T20-T28, and T30–32. ICDPIC-R recognizes only these codes in the calculation of injury severity from ICD-10, and also requires that the codes conclude with the letter “A” (indicating an initial encounter).

Mapping of ICD-10-CM codes to AIS severity can be performed in several ways, as described below.

### “GEM” mapping methods for ICD-10-CM codes

ICD-10-CM codes are first mapped to ICD-9-CM codes using the General Equivalency Mapping (GEM) tables provided by the Centers for Medicare and Medicaid Services (CMS), and then those ICD-9-CM codes are mapped to AIS using the table inherited from the Stata version of ICDPIC. The user is given the option to ignore ICD-10-CM codes if desired. Otherwise, if the GEM maps an ICD-10-CM code to two or more ICD-9-CM codes associated with different severities, the user is given the option whether to assign the greater or lesser of these severities (“GEMmax” or “GEMmin”). When the GEM maps an ICD-10-CM code to two or more ICD-9-CM codes associated with different AIS body regions, the verbal description of the ICD-10-CM code in the GEM table is used to assign a body region.

The GEM mapping method may be preferable when injuries have been recorded with a mix of ICD-9-CM and ICD-10-CM codes.

### “ROCmax” mapping method for ICD-10-CM codes

For each NTDB subject *k* with 1 to *N*_*k*_ valid ICD-10 injury codes, each valid ICD-10 code was given a score of 0 if the subject survived and a score of 1/*N*_*k*_ if the subject died (as recorded either in the Emergency Department file or in the Discharge file). For each ICD-10 code, a measure of effect (*E*) for the corresponding injury diagnosis was derived as the mean score for subjects with this code. Different cutpoints for *E* were investigated to determine how this measure could best be translated into an AIS severity. AIS body regions were assigned using the proposed CDC classification (Annest et al., 2014). For each combination of cutpoints, an ISS was calculated, and its ability to discriminate patients who survived from those who did not was determined using a C-statistic. Among the combinations resulting in a maximum C-statistic (to two decimal places), the combination was chosen that most closely approximated the ISS that had been calculated by hospital trauma registrars (*ISSAIS*).

The ROCmax method may be preferable when injuries have been recorded using ICD-10-CM, subjects are similar to those in NTDB, and the user wishes to maximize the predictive power of ISS. After some data transformation, it could be adapted to ICD-10 data formats other than ICD-10-CM. It is the default mapping method for ICD-10-CM data in ICDPIC-R.

For any of the ICD-9 or ICD-10 mapping methods in ICDPIC-R, the maximum AIS Severity for each AIS body region (*MXAISBR1* … *MXAISBR6* in the output) is 0 if there are no valid injury codes for that body region. It is recorded as “missing” if there are valid codes for that body region, but their severity cannot be determined. Otherwise, it is the maximum known severity (1 through 6) for that body region. Maximum AIS Severity (*MAXAIS* in the output data) is the maximum of (*MXAISBR1* … *MXAISBR6*); *MAXAIS* will thus be 0 if there is no diagnosis code associated with an AIS severity.

Injury Severity Score (*RISS*) is calculated according to the classic description of Baker and colleagues (Baker et al., 1974), namely the sum of the squares of the three largest elements of (*MXAISBR1* … *MXAISBR6*). The user can choose whether to assign *RISS* = 75 when any injury is assigned a severity of 6, or to reassign a severity of 5 to these injuries and calculate *RISS* as above. The latter option is the default in ICDPIC-R, since by definition (Committee on Medical Aspects of Automotive Safety, AMA, 1971) an AIS severity of 6 should denote an injury that is uniformly fatal and thus should rarely be found in hospital data.

For cases coded using ICD-10-CM, *RISS* (obtained from ICDPIC-R using the methods described) and *ISSAIS* (calculated by NTDB from AIS codes submitted by hospital trauma registrars) were compared after being categorized as recommended by Copes et al. (Copes et al., 1988) Observed mortality for each category of each method was compared. Performance of the various ISS methods was further evaluated using a C-statistic (Stata command *roctab*). The C-statistic, also known as the area under a receiver-operator characteristic (ROC) curve, ranges from 0.5 (no discrimination) to 1.0 (perfect discrimination).

For NIS data, the injury mechanism diagnoses “I10_ECAUSE1” - “I10_ECAUSE4” were renamed “I10_DX31” - “I10_DX34” so that they could be processed by ICDPIC-R along with the other diagnoses (coded as “I10_DX1” - “I10_DX30”). Observed mortality for each category of *RISS* was analyzed as for NTDB.

## Results

Among 917,865 patients recorded in the NTDB research data set for Admission Year 2015, ICD-10-CM codes were used in 92,168 cases (10.0%), of which 86,878 cases (9.5%) had from 1 to 48 valid ICD-10-CM injury codes by the criteria described above.

After development and debugging, ICDPIC-R gave results identical to ICDPIC in Stata when ICD-9-CM data were used (before the minor revisions described above).

For implementation of the ROCmax mapping method for the 86,878 cases with valid ICD-10-CM diagnoses the best combination of cutoff points for assigning each ICD-10 code an AIS severity based on its measured effect (*E*) was AIS = 1 for 0 < = *E* < .009, AIS = 2 for .009 < = *E* < .019, AIS = 3 for .019 < = *E* < .044, AIS = 4 for .044 < = *E* < .062, AIS = 5 for .062 < = *E* < 1, and AIS = 6 for *E* = 1.

For the 86,878 cases with valid ICD-10-CM codes, all methods produced an ISS for which increasing values were associated with increasing mortality (Table 1). However, 6240 cases (7.2%) did not have a recorded ISS (*ISSAIS*) in NTDB. The GEM methods were unable to categorize 1825 cases (2.3%), while the ROCmax method was able to categorize all but 6 cases (0.01%). C-statistics were calculated for the ability of each form of ISS to distinguish NTDB patients with valid ICD-10-CM diagnoses (after excluding cases that could not be categorized) who would subsequently die or not die in the hospital. For *ISSAIS,* the C-statistic was 0.81; for the GEMmax and GEMmin methods, the C-statistics were 0.75; and for the ROCmax method, the C-statistic was 0.85.

**Table 1:** For 86,878 patients in NTDB whose injuries were coded using ICD-10-CM, observed mortality(%) for categories of Injury Severity Score (ISS) using the methods described: Recorded by NTDB trauma registrars (*ISSAIS*), or estimated by ICDPIC-R (GEMmax, GEMmin, and ROCmax). For 66,417 patients in NIS, observed mortality for the last three of these categories

	*ISSAIS*	GEMmax	GEMmin	ROCmax
Calculated ISS	National Trauma Data Bank			
1–8	1.1	1.5	1.6	0.8
9–15	2.4	3.0	3.1	3.0
16–24	6.4	5.0	6.3	8.5
25–40	27.4	22.7	31.0	29.0
41–49	40.4	34.6	41.8	49.8
50–75	62.1	54.5	53.6	65.5
Unknown	4.9	2.2	2.2	0.0
Calculated ISS	National Inpatient Sample			
1–8		0.7	0.8	0.8
9–15		2.1	2.1	2.4
16–24		4.5	5.8	7.3
25–40		16.7	25.0	16.5
41–49		24.3	20.0	23.5
50–75		33.3	32.3	36.8
Unknown		2.5	2.5	0.0

Categorized *RISS* and *ISSAIS* are compared in Tables 2, 3 and 4, after excluding cases without a recorded *ISSAIS* in NTDB. The GEMmax method categorized cases into the same category as *ISSAIS* 69% of the time, and into the same or an adjacent category 94% of the time. The GEMmin method categorized cases into the same category as *ISSAIS* 72% of the time, and into the same or an adjacent category 95% of the time. The ROCmax method categorized cases into the same category as *ISSAIS* 59% of the time, and into the same or an adjacent category 92% of the time.

**Table 2:** For 80,638 patients in NTDB whose injuries were coded using ICD-10-CM and who had been registered with an Injury Severity Score *(ISSAIS)*, the percentages who were categorized as shown into ISS categories by *ISSAIS* and by ICDPIC-R (*RISS*, using the GEMmax method)

*RISS*, GEMmax Method							
	1–8	9–15	16–24	25–40	41–49	50–75	Uncoded
*ISSAIS*							
1–8	44.1	3.6	1.3	0.1	0.0	0.0	2.0
9–15	6.2	17.7	8.5	0.5	0.0	0.0	0.1
16–24	0.7	1.4	5.3	1.8	0.1	0.0	0.1
25–40	0.3	0.4	2.1	1.9	0.3	0.2	0.1
41–49	0.0	0.0	0.0	0.2	0.1	0.1	0.0
50–75	0.0	0.0	0.0	0.1	0.1	0.2	0.0

**Table 3:** For 80,638 patients in NTDB whose injuries were coded using ICD-10-CM and who had been registered with an Injury Severity Score *(ISSAIS)*, the percentages who were categorized as shown into ISS categories by *ISSAIS* and by ICDPIC-R (*RISS*, using the GEMmin method)

*RISS*, GEMmin Method							
	1–8	9–15	16–24	25–40	41–49	50–75	Uncoded
*ISSAIS*							
1–8	45.1	3.7	0.4	0.1	0.0	0.0	2.0
9–15	8.1	19.6	5.1	0.2	0.0	0.0	0.1
16–24	1.0	2.0	5.4	1.1	0.0	0.0	0.1
25–40	0.3	0.5	2.3	2.0	0.1	0.1	0.0
41–49	0.0	0.0	0.1	0.2	0.1	0.1	0.0
50–75	0.0	0.1	0.0	0.2	0.1	0.1	0.0

**Table 4:** For 80,638 patients in NTDB whose injuries were coded using ICD-10-CM and who had been registered with an Injury Severity Score *(ISSAIS)*, the percentages who were categorized as shown into ISS categories by *ISSAIS* and by ICDPIC-R (*RISS*, using the ROCmax method)

*RISS*, ROCmax Method							
	1–8	9–15	16–24	25–40	41–49	50–75	Uncoded
*ISSAIS*							
1–8	41.5	7.4	1.1	1.1	0.0	0.0	0.0
9–15	15.6	12.3	4.2	1.1	0.0	0.0	0
16–24	2.3	2.6	3.6	1.0	0.1	0.0	0
25–40	0.6	0.8	2.2	1.6	0.1	0.1	0
41–49	0.0	0.1	0.1	0.2	0.1	0.0	0
50–75	0.0	0.0	0.1	0.2	0.1	0.1	0

One limitation in comparing *RISS* to *ISSAIS* became apparent when discrepant cases were restricted to those with a single diagnosis. Theoretically, *ISSAIS* in these cases should only be the square of a number from 1 to 5, but among these 5190 cases there were 1591 (30.7%) for whom this was not true. Furthermore, the recorded *ISSAIS* was highly variable for a given diagnosis; the most common diagnosis codes resulting in discrepancy between *RISS* and *ISSAIS* are shown in Table 5.

**Table 5:** For NTDB patients with a single valid ICD-10-CM diagnosis, the ten diagnoses that were most frequently classified into different categories by *ISSAIS* and by ICDPIC-R (*RISS*, using the ROCmax method). SDH = Subdural hemorrhage; SAH = Subarachnoid hemorrhage; LOC = Loss of consciousness; Fx = Fracture, Mod = Moderate; U = Uncoded

ICD-10-CM and brief description	*RISS*	*ISSAIS* category						
		1–8	9–15	16–24	25–40	41–49	50–75	U
S06.5X0A SDH without LOC	16	6	78	91	52			
S06.6X0A SAH without LOC	9	68	17	5	1			
S22.42XA Multiple left rib Fxs	16	16	58	7				
S02.0XXA Closed Fx skull vault	4	52	44	14	7		1	
S22.41XA Multiple right rib Fxs	16	14	48	6	1			
S27.0XXA Pneumothorax	9	30	9	11	4		1	1
S06.5X9A SDH, unspecified LOC	16	1	13	22	18			
S06.6X9A SAH, unspecified LOC	9	19	7	3				
S36.031A Mod laceration of spleen	16	1	16	12	3			
S02.10XA Unspecified Fx skull base	9	2	48	14	3			

In the NIS data set for the Fourth Quarter of 2015, a principal ICD-10-CM diagnosis of trauma by the criteria described above was present in 66,417 patients, who had from 1 to 30 listed diagnoses plus up to 4 injury mechanism diagnoses. All methods in ICDPIC-R produced an ISS for which increasing values were associated with increasing mortality (Table 1). The GEM methods were unable to categorize 1577 cases (2.4%), while the ROCmax method was able to categorize all but 99 cases (0.15%). For the GEMmax method, the C-statistic was 0.75; for the GEMmin method, the C-statistic was 0.76; and for the ROCmax method, the C-statistic was 0.78.

## Program implementation

In order to implement ICDPIC-R, the user must first install the free programming language R. This can be done by accessing http://www.r-project.org and following the link to download R. Further instructions and troubleshooting help are available at this website and elsewhere. Users may also wish to install the adjunctive program “R Studio” from http://www.rstudio.com/products/RStudio, but this is not necessary.

Basic procedures for issuing commands in R are available in many locations, or may be obtained from a colleague already familiar with the program. In the instructions below, it should be noted that the result of a command can be assigned a name using the “<-” operator, and that R will recognize a forward-slash (“/”) or double back-slash (“\\”), but not a single back-slash (“\”).

Once R has been installed and is working properly, the following commands will install and load ICDPIC-R:

**install.packages(“devtools”)**

**devtools::install_github(“ablack3/icdpicr”)**

**library(icdpicr)**

During the installation of packages, the user may be prompted to select a “CRAN Mirror”; it does not matter which is selected. Once the packages have been successfully installed, further documentation about ICDPIC-R can be obtained by the command.

**help(package = icdpicr)**

The input data file must be in CSV (comma-separated variables) format with each row corresponding to one person. The names for the fields containing diagnosis codes (ICD-9 and/or ICD-10) must begin with the same prefix (e.g., *dx1*, *dx2*, …). There is no maximum number of diagnosis codes per person. The following command will load the data into R:

**data.in < − read.csv(“C:/path/to/input.csv”, stringsAsFactors = F)**

Once the data file has been successfully loaded, the “categorize trauma” procedure can be run with the following command:

**data.out < − cat_trauma(data.in,“dx”)**

This procedure will return the original data with additional fields for the AIS region and severity assigned to each anatomic injury code, *MAXAIS*, *MXAISBR1* … *MXAISBR6*, *RISS*, and CDC mechanism category. If not otherwise specified, the ROCmax method will be used for ICD-10-CM data. To use the GEM methods, the command should be modified as follows:

**data.out < − cat_trauma(data.in,“dx”, i10_iss_method = “gem_max”)**

or

**data.out < − cat_trauma(data.in,“dx”, i10_iss_method = “gem_min”)**

To export the resulting table in CSV format (e.g., for subsequent analysis using more familiar software) enter the following command:

**write.csv(data.out, “C:/path/to/output.csv”, row.names = F)**

If the user is interested in viewing the tables used for mapping, they may be found at https://github.com/ablack3/icdpicr/tree/master/lookup_tables. Users may wish to copy them for use with another program if they find that easier than working in R.

If the user would prefer to avoid any programming, a web application has also been provided at https://ablack3.shinyapps.io/icdpicr_app. This implements the current version of ICDPIC-R using only a simple point-and-click interface, but is limited to datasets with fewer than approximately 10,000 records.

## Discussion

The “gold standard” for injury severity scoring obviously involves the judgment of an experienced clinician and/or registrar, and access to the actual subject or the primary medical record. However, the time and effort required to approach this ideal is only possible in specialized settings and for subjects who can be thoroughly evaluated. We therefore hope that these methods to derive approximate scoring using administrative data will be useful for population-based studies.

ISS coding among trauma registries contributing to the NTDB during the last quarter of 2015 was quite variable, even for patients with a single diagnosis (Table 5). This might be expected given that ICD-10-CM coding had just been introduced in American hospitals. The imprecision is likely to improve as the new coding format becomes more familiar, but suggests that some standardized method of deriving a severity score from individual diagnoses may be preferable for large centralized registries.

As might also be expected, the ROCmax method did not produce as large a C-statistic in the validation sample (NIS) as it had in the development sample (NTDB). Nevertheless, it was the best of the three ICDPIC-R methods in this respect. For a given estimate of ISS, observed mortality was lower in NIS than in NTDB (Table 1), but the latter probably includes many Emergency Department deaths that would have been excluded from the NIS (Phillips et al., 2008).

Version 0.1.0 of ICDPIC-R undoubtedly will have unexpected practical deficiencies, which users are encouraged to identify and communicate to the authors. If the CDC update of the Barell Matrix for ICD-10-CM codes (Hedegaard et al., 2016) is eventually associated with AIS severity estimates in the public domain, these could be incorporated into future versions of ICDPIC-R in place of or in addition to the methods described above. Similarly, the AAAM mapping, or mappings developed in other countries, could be incorporated if they are made available in the public domain.

In any event, a new version of ICDPIC-R is planned after the next release of NTDB and NIS, which should each contain a full year of data coded using ICD-10-CM. The additional data should allow additional precision, and perhaps enable a more robust algorithm to build on the ROCmax mapping strategy. Other reference databases may become available, and the GEM table can also be updated to the latest version published by CMS. Based upon previous user comments about the Stata version of ICDPIC, Version 0.1.0 of ICDPIC-R does not include modules for comorbidity scores, AP, NISS, TRISS, ASCOT, ICISS, TMPM, or Barell Matrix (or its ICD-10 successor). However, these functions could be added in subsequent versions if there is sufficient demand.

## Abbreviations

AAAM: Association for the Advancement of Automotive Medicine
ACS: American College of Surgeons
AHRQ: Agency for Healthcare Research and Quality
AIS: Abbreviated Injury Scale
AMA: American Medical Association
AP: Anatomic Profile
ASCOT: A Severity Characterization of Trauma
CDC: Centers for Disease Control and Prevention
CMS: Centers for Medicare and Medicaid Services
CSV: Comma-Separated Variables
ESP: Estimated Survival Probability
FHM: Fractional Hospital Mortality
GEM: General Equivalency Mapping
ICD: International Classification of Diseases
ICD-10: ICD, 10th Revision
ICD-10-CM: ICD-10, Clinical Modification
ICD-8: ICD, 8th Revision
ICD-9: ICD, 9th Revision
ICD-9-CM: ICD-9, Clinical Modification
ICDA: ICD, Adapted
ICDPIC: ICD Programs for Injury Categorization
ICDPIC-R: ICDPIC, R version
ICISS: ICD-9-based Injury Severity Score
ISS: Injury Severity Score
ISSAIS: ISS calculated by NTDB registrars
MAXAIS: Maximum AIS
MXAISBR1…MXAISBR6: Maximum AIS for body regions 1…6
NIS: National Inpatient Sample
NISS: New Injury Severity Score
NTDB: National Trauma Data Bank
RISS: ISS calculated using ICDPIC-R
ROC: Receiver-Operator Characteristic
TMPM: Trauma Mortality Prediction Model
TRISS: Trauma and Injury Severity Score
